# Editorial: Tumor-host interactions: metabolic and signaling pathways altered in cancer, immune and stromal cells

**DOI:** 10.3389/fmolb.2026.1778371

**Published:** 2026-01-09

**Authors:** Joanna Kopecka, Valentina Audrito

**Affiliations:** 1 Department of Oncology, University of Torino, Torino, Italy; 2 Department of Science and Technological Innovation, University of Eastern Piedmont, Alessandria, Italy

**Keywords:** cancer cells, immune cells, stromal cells, TME, tumor microenvironment, tumor progression, tumor-host crosstalk

Tumor-host interactions within the tumor microenvironment (TME) represent a critical area of cancer research. The TME is characterized by hypoxia, nutrient competition, acidic pH, chronic inflammation, and dynamic communication between cancer, immune, and stromal cells. These interactions involve the release of soluble factors such as metabolites, cytokines, growth factors, and exosomes, which drive aberrant signaling pathways and phenotypic and metabolic plasticity in both tumor and immune cells (De Visser and Joyce, 2023) This metabolic adaptation is crucial for tumor growth, metastatic dissemination, and therapy resistance. Recent studies have highlighted the potential of targeting altered signaling and tumor metabolism as a promising approach for the development of new cancer therapies (Bejarano et al., 2021; Dominiak et al., 2020). However, a comprehensive understanding of the molecular and biochemical mechanisms by which cancer and immune/stromal cells reprogram their metabolism and exchange metabolites, and soluble factors is still lacking. This gap in knowledge underscores the need for further investigation into the metabolic and signaling pathways within the TME to develop new anti-cancer targets and improve therapeutic outcomes.

This Research Topic includes four original and review papers addressing the complex dynamics of tumor-host interactions, elucidating the molecular and biochemical mechanisms underlying the metabolic and signaling crosstalk between cancer, immune, and stromal cells within the TME, offering novel potential treatment strategies.

The first study by Hafiz Muhammad Faraz Azhar et al. addresses hypoxia-driven signaling by focusing on hypoxia-inducible factor-1 (HIF-1), a master regulator of metabolic adaptation in the TME. Using a Boolean Regulatory Network based on the René Thomas qualitative modeling framework, the authors dissected the regulatory interactions between HIF-1 and key signaling and metabolic nodes, including VEGF, ERK, AKT, GLUT-1, β-catenin, C-MYC, OGT, and p53. Their simulations revealed that tightly regulated cyclic activation of p53, β-catenin, and AKT maintains a homeostatic or recovery state, whereas disruption of this balance drives pathological cancer states. Notably, VEGF overexpression promoted ERK and GLUT-1 upregulation, linking angiogenesis, glucose metabolism, and aggressive tumor behavior. These findings highlight how hypoxia-dependent signaling rewires tumor metabolism and supports cancer progression within the TME. De Carvalho Freitas Ramos et al., provided experimental evidence that targeting metabolic vulnerabilities can suppress tumor growth and dissemination. The authors demonstrated that curcumin induces reactive oxygen species (ROS)–mediated apoptosis in oral squamous cell carcinoma (OSCC) using 2D cultures, 3D spheroids, and *in vivo* mouse models. Curcumin disrupted tumor spheroid integrity, reduced tumor growth, and significantly decreased tumor emboli and metastatic nodules, underscoring its ability to counteract TME-associated therapy resistance. Together, these findings suggest that curcumin could be considered a promising candidate for OSCC therapy, a cancer type that is often refractory to current treatment strategies. In the brief research paper by Du et al., the authors explored the role of Krüppel-like factor 14 (KLF14) and its underlying mechanism(s) of action in cell-cycle regulation in cervical cancer. KLF14, a member of the Krüppel-like transcription factor family (KLFs), acts as a key regulator of tumor pathogenesis. Throught a lentivirus infection leading to stable overexpression of KLF14 the authors identified that this factor induces S-phase arrest in cervical cancer cells and inhibited the proliferation of cervical cancer cells *in vivo*. The induction of S-phase arrest was related to its zinc-finger structure. Moreover, KLF14 also activates the JNK pathway to induce S-phase arrest and promote the expression of cyclin-dependent kinase 2 (*CDK2*) and cyclin A2 (CCNA2). Therefore, this study opened the way for future investigation on KLF14 manipulation for cancer therapy. Lastly, the review paper by Hernández-Magaña et al., addressed the emergence of rationally designed cancer therapies that target cell plasticity mechanisms, particularly those involved in epithelial–mesenchymal transition (EMT). These strategies aim to block the transformation of epithelial cells into cancerous phenotypes. The authors focused on hepatocellular carcinoma (HCC) and reviewed engineering principles that could be applied to the rational design of personalized therapeutic approaches. They described multiple HCC features associated with phenotypic plasticity across different biological levels and supported the development of models that accurately represent EMT dynamics. The conceptualization of such models may enable the application of engineering principles to design and optimize cancer treatments, highlighting emerging research areas such as systems biology.

In conclusion, the studies collected in this Research Topic underscore the pivotal role of metabolic and signaling plasticity in tumor–host interactions within the TME. By combining computational, experimental, and conceptual approaches, these studies provide complementary insights into how hypoxia, metabolic rewiring, cell-cycle regulation, and phenotypic plasticity drive cancer progression and therapeutic resistance. Together, they highlight the importance of integrative and interdisciplinary strategies to better understand TME complexity and to advance the development of more effective and personalized anti-cancer therapies.
